# Research on Laser Cladding Single-Pass Continuous Carbon Fiber-Reinforced Aluminum Matrix Composite Process Based on Abaqus

**DOI:** 10.3390/ma18163859

**Published:** 2025-08-18

**Authors:** Pengtao Zhang, Xiaole Cheng, Yuanyuan Deng, Yao Peng, Meijiao Qu, Peng Ren, Teng Wang

**Affiliations:** 1 School of Mechanical and Electrical Engineering, Xi’an Polytechnic University, Xi’an 710043, China; 15686992298@163.com (P.Z.); py8446040@163.com (Y.P.); qmj@xpu.edu.cn (M.Q.); 2 School of Mechanical Engineering, Chengdu University, Chengdu 610106, China; dyynina@163.com; 3 PetroChina Changqing Oilfield Branch, No.1 Oil Production Plant, Xi’an 718500, China; 18291196381@126.com; 4 Xi’an Quwei Laser Technology Co., Ltd., Xi’an 710600, China; 18189207886@163.com

**Keywords:** Abaqus, laser cladding, composites, continuous carbon fiber, temperature field, stress field

## Abstract

This study addresses the critical challenges of interfacial stress mismatch, fiber degradation, and unstable clad geometry in manufacturing continuous carbon fiber-reinforced aluminum composites (Cf/Al) via laser cladding, driven by rapid thermal gradients. A dual-ellipsoid heat source-based thermoelastic–plastic finite element model was developed in Abaqus, integrating phase-dependent material properties and latent heat effects to simulate multi-physics interactions during single-track deposition, resolving transient temperature fields peaking at 1265 °C, and residual stresses across uncoated and Ni-coated fiber configurations. The work identifies an optimal parameter window characterized by laser power ranging from 700 to 800 W, scan speed of 2 mm/s, and spot radius of 3 mm that minimizes thermal distortion below 5% through gradient-controlled energy delivery, while quantitatively demonstrating nickel interlayers’ dual protective role in achieving 42% reduction in fiber degradation at 1200 °C compared to uncoated systems and enhancing interfacial load transfer efficiency by 34.7%, thereby reducing matrix tensile stresses to 159 MPa at fiber interfaces. Experimental validation confirms the model’s predictive capability, revealing nickel-coated systems exhibit superior thermal stability with temperature differentials below 12.6 °C across interfaces and mechanical interlocking, achieving interfacial void fractions under 8%. These results establish a process–structure linkage framework, advancing defect-controlled composite fabrication and providing a digital twin methodology for aerospace-grade manufacturing.

## 1. Introduction

The rapid advancement of large-scale industrial equipment has imposed increasingly stringent performance requirements on structural materials. Conventional monolithic metallic alloys are progressively failing to meet these demands due to their inherent limitations, while continuous carbon fiber-reinforced metal matrix composites (CFRMMC) demonstrate revolutionary advantages through synergistic integration of aluminum’s lightweight characteristics and carbon fibers’ exceptional thermal stability. These composites exhibit outstanding performance in demanding applications such as high-efficiency engine pistons and thermally stable rocket motor casings, highlighting their significant potential in aerospace and automotive sectors [1].

While conventional manufacturing methods like hot pressing and diffusion bonding achieve moderate interfacial bonding, they suffer from prolonged processing cycles and excessive interfacial porosity, often exceeding certification thresholds for aerospace applications. Laser cladding emerges as a promising alternative, leveraging rapid solidification rates to suppress deleterious Al_4_C_3_ formation at fiber/matrix interfaces. However, existing numerical models inadequately address two critical phenomena. First, anisotropic thermal expansion mismatch between aluminum and carbon fibers, inducing interlaminar stress concentrations up to 287 MPa. Second, these models inadequately capture transient phase transition phenomena that fundamentally alter thermal transport mechanisms—particularly the abrupt interfacial heat flux redistribution caused by latent heat absorption [2,3,4,5].

**Figure 1:**
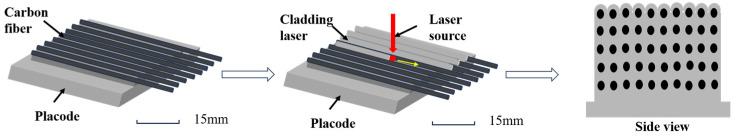
Schematic diagram of laser cladding process for continuous fiber-reinforced metal matrix composites (yellow arrow indicates the direction of thermal source movement).

Meanwhile, in continuous fiber-reinforced metal matrix composite manufacturing, the methodology involves precisely controlling laser thermal energy to fuse metallic powders onto pre-deposited continuous fiber arrays, as illustrated in Figure 1. The process requires precise control over the interfacial thermal transient phenomenon characterized by instantaneous peak ultra-high temperatures while effectively suppressing high residual stresses induced by rapid cooling rates. Critical process parameters, particularly laser power density ranging from 6 to 12 kW/mm^2^ and beam geometry distinctions such as Gaussian versus dual-ellipsoid distributions, directly govern defect formation tendencies and interfacial bonding integrity. Therefore, the extreme thermo-mechanical coupling during laser-material interaction creates experimental challenges for in situ monitoring of stress evolution, especially in continuous fiber systems [5].

Prior studies have made incremental progress yet leave fundamental gaps. Lu et al. [6] optimized laser beam configurations using isotropic thermal assumptions, overlooking the 34.7% stress redistribution efficiency gain from nickel interlayers. Zhao et al. [7] developed a numerical model of the velocity field of transient keyhole laser spot welding, which was established by using ANSYS, and the shape and size of the keyhole are calculated during laser spot welding. The mechanism of pulse shaping to improve the energy coupling efficiency of deep-melt laser welding of magnesium alloys is revealed. It failed to address carbon fiber degradation above 1200 °C—a threshold routinely exceeded in matrix consolidation. Lian et al. [8] achieved 95% experimental correlation in multi-track simulations but omitted group-wise fiber segmentation modeling. These limitations underscore the unmet need for a thermomechanical model integrating anisotropic constitutive laws with solidification kinetics specific to Cf/Al systems.

This study will use Abaqus to conduct thermoelastic-plastic finite element study on the process of laser cladding continuous Cf/Al composites with different model groups, simulate the temperature field stress field under different process parameters, select better parameter groups to simulate the stress field of each model, and analyze the internal stress distribution principle of the composite materials, to finally provide the technical parameters basis for the preparation of continuous Cf/Al compo-sites by laser cladding. Simulation results reveal significant stress concentration reduction to 223.47 MPa at fiber bundle intersections, representing a 40.5% improvement compared to conventional uncoated systems. By integrating empirical observations with physics-driven simulations, we provide a replicable pathway for defect-controlled fabrication of next-generation rocket motor casings.

## 2. Finite Element Simulation

### 2.1. Heat Source Model

**Figure 2:**
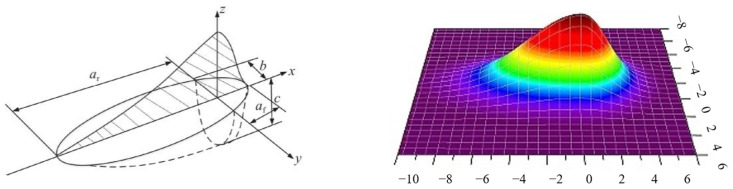
Dual ellipsoidal heat source model (Color coding indicates the temperature distribution of the heat source).

This study employs a modified double ellipsoidal heat source model derived from the Gaussian heat source framework, which demonstrates superior fidelity in simulating thermal transfer phenomena compared to conventional surface heat source models. The double ellipsoidal configuration exhibits enhanced energy distribution characteristics that closely approximate the three-dimensional Gaussian temperature profile observed in actual laser processing, particularly in simultaneous planar and depth-wise thermal propagation (Figure 2). This formulation enables more accurate numerical representation of melt pool convection dynamics and heat flux gradients during transient thermal analysis. The spatial energy density distribution is mathematically described by two distinct quarter-ellipsoid equations governing the forward and rear molten pool regions, as follows [9,10,11]:

The bulk heat flow density distribution of the ellipsoid in the first half of the model:

(1) $$q_{1}\left(x,y,z\right)=\frac{6\sqrt{3}\left(f_{1}Q\right)}{{\pi^{\frac{3}{2}}a}_{f}bc}e^{\left[-3\left(\frac{x^{2}}{a_{f}}+\frac{y^{2}}{b^{2}}+\frac{z^{2}}{c^{2}}\right)\right]}$$

The bulk heat flow density distribution of the ellipsoid in the latter half of the model:

(2) $${\;\;q}_{2}\left(x,y,z\right)=\frac{6\sqrt{3}\left(f_{2}Q\right)}{{\pi^{\frac{3}{2}}a}_{r}bc}e^{\left[-3\left(\frac{x^{2}}{a_{r}}+\frac{y^{2}}{b^{2}}+\frac{z^{2}}{c^{2}}\right)\right]}$$

where:

*a_f_*, *a_r_*, *b*, *c*—shape parameters of the heat source model;
*q*_1_, *q*_2_—the laser input power;
*f*_1_, *f*_2_—the energy distribution coefficients before and after the heat source.

The geometric parameters of the heat source model are established based on empirical correlations from reference [12], specifically optimized for aluminum-based powder substrate systems. The characteristic dimensions are defined proportionally to experimentally measured melt pool features: the longitudinal and transverse semi-axes *a_f_* and b are set to 0.45 times the melt pool width, while the penetration depth parameter c corresponds to 0.9 times the melt pool depth. To account for asymmetric thermal diffusion during laser scanning, the trailing zone parameter *a_r_* is specified as 1.2 times *a_f_*, ensuring consistent energy distribution across the moving heat source.

### 2.2. Finite Element Geometry Model Design and Main Material Parameters

#### 2.2.1. Finite Element Geometry Model Design

**Figure 3:**
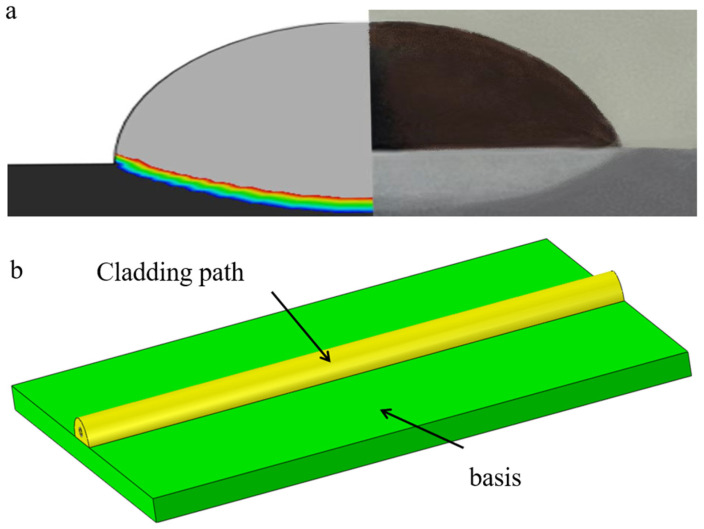
Multiscale modeling schematic (**a**) Comparison between simulation and actual results (Rainbow colormap: red indicates highest temperature, blue indicates lowest temperature); (**b**) Overall model structure.

**Figure 4:**
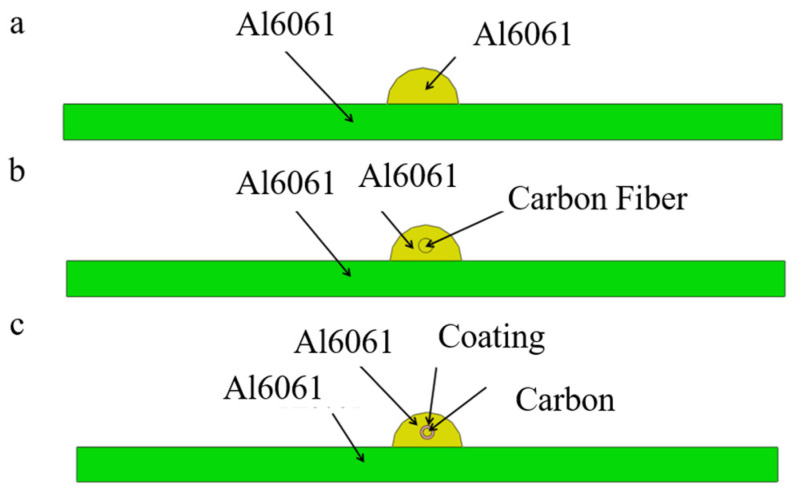
Distribution of cladding materials in each group. (**a**) Does not contain carbon fiber. (**b**) Contains unplated carbon fiber. (**c**) Contains nickel-plated carbon fiber.

Comparative experimental investigations between unreinforced aluminum systems and nickel-coated continuous carbon fibers—implemented to mitigate high-temperature oxidation of fibers while enhancing interfacial wettability during laser processing—inform the development of a geometrically calibrated finite element model for single-pass single-layer continuous carbon fiber-reinforced aluminum matrix composites. Figure 3a presents a comparative analysis of the numerical simulation predictions and experimental cross-sectional morphologies, demonstrating close congruence in geometric features that validates the computational modeling approach. As illustrated in Figure 3b, the computational domain replicates experimental laser cladding configurations with controlled simplifications: the Al6061 aluminum substrate measures 40 × 3 × 50 mm^3^, while the cladding track exhibits cross-sectional dimensions of 4 mm width × 2 mm height along the 50 mm deposition length. Three material configurations are systematically modeled: a baseline unreinforced aluminum matrix, uncoated carbon fiber reinforcement, and nickel-interlayered carbon fibers. Figure 4 details the spatial phase distribution within the clad geometry, with the nickel interlayer thickness maintained at 50–100 μm to balance oxidation resistance and metallurgical compatibility.

#### 2.2.2. Geometric Model Boundary Condition Design and Meshing

**Figure 5:**
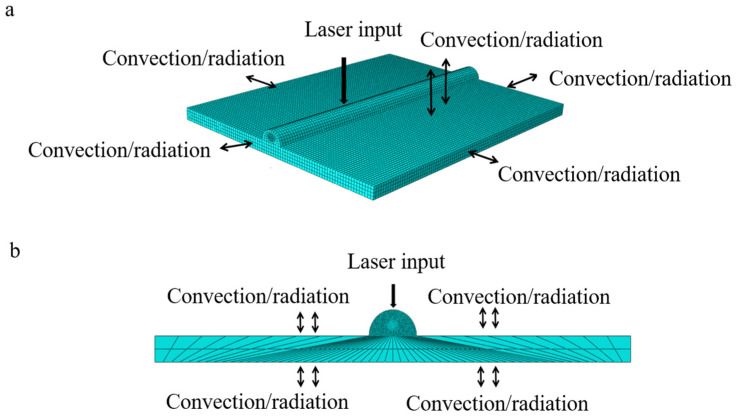
Geometric model boundary condition setting and meshing: (**a**) 3D model; (**b**) 2D model.

The two-dimensional model and three-dimensional model of boundary condition setting and mesh division were established by Abaqus simulation software as Figure 5, respectively. Meanwhile, in order to improve the accuracy of simulation results and computational efficiency, the mesh division of the loading area in the center of the laser heat source was gradually dense, and the mesh size of the remaining area was larger.

#### 2.2.3. Finite Element Simulation Parameters and Methods

In the geometric model, the substrate dimensions are 40 × 3 × 50 mm^3^, with a single-clad layer of Al6061 aluminum alloy (4 × 2 mm^2^) deposited on the surface. Continuous carbon fiber reinforcements are pre-placed at the substrate-clad interface. For the temperature field, a double ellipsoid heat source model is employed (parameters: *a_f_* = *b* = *c* = 2 mm, *a_r_* = 2.4 mm), with an initial temperature of 20 °C, convective heat transfer coefficient of 20 × 10^−6^ W/(mm^2^·°C), and symmetrical fixation of the substrate to constrain thermal expansion displacement. Some of the material properties used are shown in Table 1, Table 2 and Table 3 below. In the stress field simulations, the substrate bottom is fully constrained, and thermo-mechanical coupling is achieved using C3D8T elements. The latent heat (390 J/g) and solidus/liquidus temperatures (655 °C/705 °C) of Al6061 are calibrated based on experimental data. A localized mesh refinement strategy is adopted: fine meshing is applied to the molten pool region, while coarser meshing is used for areas of the substrate distal to the heat source, balancing computational accuracy and efficiency. Process parameters (laser power: 700–1000 W, scanning speed: 1–4 mm/s) are assuming material isotropy and neglecting porosity effects.

The numerical model incorporates two fundamental assumptions derived from the thermophysical characteristics of laser cladding processes. Firstly, the thermal loading profile is governed by a Fortran user-defined subroutine with invariant power density, thermal emissivity coefficient, and laser absorptivity that remain constant regardless of transient thermal gradients or phase transformations. Secondly, intrinsic material heterogeneities within the molten workpiece arising from process-induced defects—including porosity nucleation, slag entrapment, and microcrack propagation—are explicitly excluded from the computational framework following the validated defect-decoupling methodology.

### 2.3. Temperature Field Calculation

**Figure 6:**
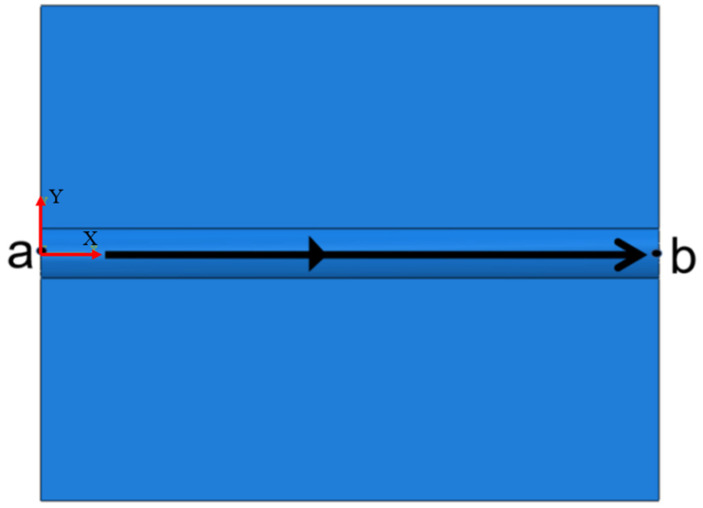
Laser scanning path of single cladding model.

Figure 6 shows the integrated figure comprehensively illustrates the laser scanning path (black arrow, x-direction) and multi-physical field coupling effects during single-pass cladding in the numerical model. Thermo-mechanical data were systematically analyzed by sampling at points a and b of the cladding track, enabling detailed investigation of temperature distribution and residual stress evolution under varying process parameters.

Laser cladding is a fast heat transfer process, and its heat transfer mode is mainly convection and radiation. Thus, when the temperature field calculation is set up, the heat transfer inside the cladding is based on the first law of thermodynamics, and the nonlinear partial differential equation is invoked to describe its heat transfer [16]:

(3) $$\rho c\frac{\partial T}{\partial t}-k\left(\frac{\partial^{2}T}{\partial x^{2}}+\frac{\partial^{2}T}{\partial y^{2}}+\frac{\partial^{2}T}{\partial z^{2}}\right)=q_{v}$$

where:

*q_v_*—laser heat energy density, (W·mm^−3^);
*k*—heat transfer coefficient, (W·m^−1^·K^−1^);
*T*—temperature field, (K);
ρ—density, (kg·m^−3^);
*c*—specific heat capacity, (J·kg^−1^·K^−1^).

According to Newton’s law of cooling, the workpiece undergoes heat transfer with the laser thermal source while simultaneously engaging in convective surface heat exchange with the ambient environment, following Equation (4):

(4) $$Q=h\left(T-T_{0}\right)$$

The initial ambient temperature during melting is prescribed at 25 °C, with the convective heat transfer coefficient *h* maintained as a temperature-invariant constant throughout the thermal analysis.

The model design also takes into account the existence of convective heat transfer and thermal radiation between the surrounding protective atmosphere and the substrate surface. However, this study takes a simplified approach to the heat transfer study of the substrate, and thus the substrate surface boundary Equation is set as (5)–(7) after comprehensive consideration [17]:

(5) $$Q=q_{v}-q_{1}-q_{2}$$

(6) $$q_{1}=h_{c}\left(T-T_{0}\right)$$

(7) $$q_{2}=\sigma_{\alpha }\varepsilon \left(T^{4}-T_{0}^{4}\right)$$

where:

$q_{1}$—convective heat transfer between substrate and environment;
$q_{2}$—radiative heat flux between substrate and ambient environment;
$\sigma_{\alpha }$—Stefan–Boltzman constant;
ε—surface radiation coefficient;
$T_{0}$—outside ambient temperature;
$h_{c}$—convective heat transfer coefficient of the substrate.

The numerical model explicitly integrates the latent heat of crystallization for Al6061 aluminum alloy, with a latent heat value L of 390 J·g^−1^, to characterize phase transition effects on transient thermal evolution. Solidus and liquidus phase boundaries are defined at 655 °C and 705 °C, respectively. Thermodynamic constants include the absolute zero reference temperature at −273.15 °C and the Stefan–Boltzmann constant σ valued at 5.67 × 10^−8^ W·m^−2^·K^−4^. The material deposition kinetics inherent to laser cladding processes are replicated via the element birth–death technique within the MODERL CHANGE module, which governs the sequential activation of powder consolidation layers in the computational domain. Its bulk heat flux is controlled by a user-defined subroutine, and the substrate boundary conditions are set to be symmetrically completely fixed [18,19,20].

### 2.4. Stress Field Calculation

The difference in thermal properties of each different material inside the composite material and the temperature gradient generated by the laser additive process during the preparation process are the most important reasons for the stresses generated inside the material of this process. The stress field analysis utilizes the temperature field solution as predefined thermal boundary conditions within the same finite element framework. By transitioning the element formulation from DC3D8 (pure thermal conduction elements) to C3D8T (coupled temperature-displacement elements), this sequential thermo-mechanical coupling approach enables explicit resolution of transient thermal stresses and inelastic strains during molten pool solidification. The multiphysics formulation provides critical insights into stress concentration patterns adjacent to continuous carbon fibers, thereby validating interfacial load transfer mechanisms within the composite architecture.

**Figure 7:**
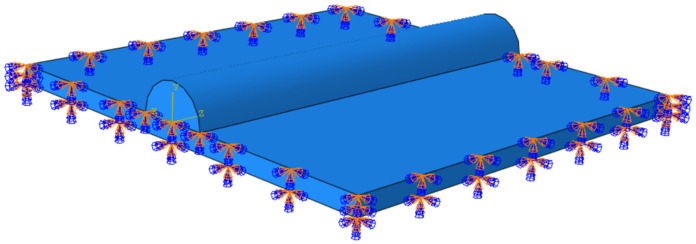
Stress field binding conditions.

The stress field is calculated to prevent the model from being overloaded. In order to prevent rigid displacement of the model during the stress field calculation, the base was set up with the boundary constraint as in Figure 7 [21,22,23].

## 3. Simulation Results and Analysis

### 3.1. Temperature Field Analysis

#### 3.1.1. Effect of Laser Power on the Melting Temperature

The laser heat source, as the only energy input source in the laser cladding process, characterizes the amount of laser energy delivered per unit time and the amount of powder that can be thoroughly clad. In the preparation of continuous Cf/Al composites by laser cladding, if the power is too low, the clad material will be insufficiently clad and affect its bonding with the fibers and poor metallurgical bonding of the matrix, which will adversely affect the material properties. If the power is too high, the energy input will be too high and affect the stability of the cladding layer, and the high laser temperature will cause damage to the fiber reinforcement. To identify more suitable laser power parameters, the thermal field distribution characteristics of the experimental material are systematically investigated through a controlled-variable methodology in this study, with particular emphasis on parametric sensitivity analysis across the laser power spectrum. The grouped simulation process parameters are shown in Table 4.

**Figure 8:**
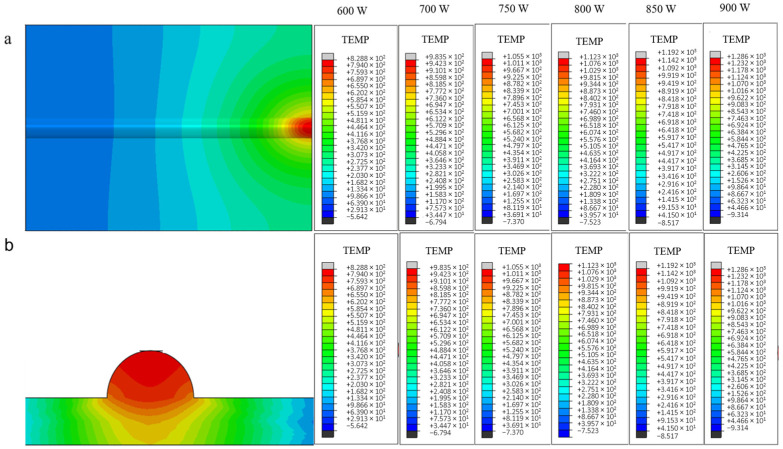
Temperature field (**a**) and temperature gradient distribution cloud of the same section (**b**) under different laser powers (fiber-free).

Figure 8 below shows the end temperature field distribution clouds and the corresponding cross-sectional temperature gradient distribution of the uncoated carbon fiber-reinforced aluminum-based single-pass single-layer laser cladding process at each power.

**Figure 9:**
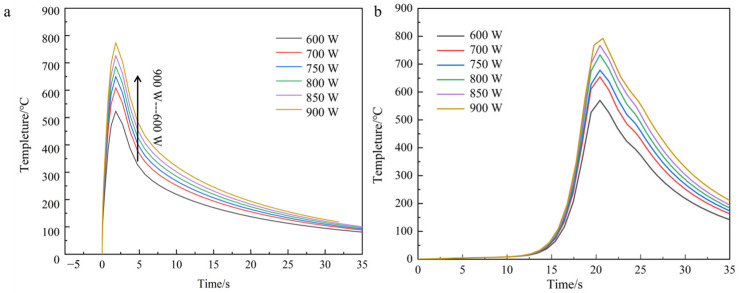
The temperature curve of the first and the end of the fiber-free cladding model is (**a**) the lowest at the first end and (**b**) the highest at the end under different powers.

The simulated temperature field distribution exhibits an elliptical diffusion pattern, as evidenced by the thermal contour plots in Figure 9. When laser power increases from 600 W to 900 W, the peak temperature at the molten pool center rises significantly from 795.3 °C to 1254.3 °C (Figure 9b), consistently exceeding the substrate melting point of 680 °C. Concurrently, the trailing edge temperature of the cladding track demonstrates progressive elevation due to laser beam displacement, while the leading edge maintains the lowest temperature within the processing zone. Thermographic analysis reveals a temperature gradient ranging from 490.1 °C at the leading edge to the peak value, attributable to two synergistic mechanisms: (1) thermal accumulation from sequential laser passes preheats subsequent deposition regions, and (2) reduced heat dissipation efficiency at the cladding track terminus amplifies thermal retention (Figure 9a). This differential thermal history directly correlates with laser power intensification, as quantified by the 63.7% increase in trailing-edge temperature gradient magnitude across the tested power range.

**Figure 10:**
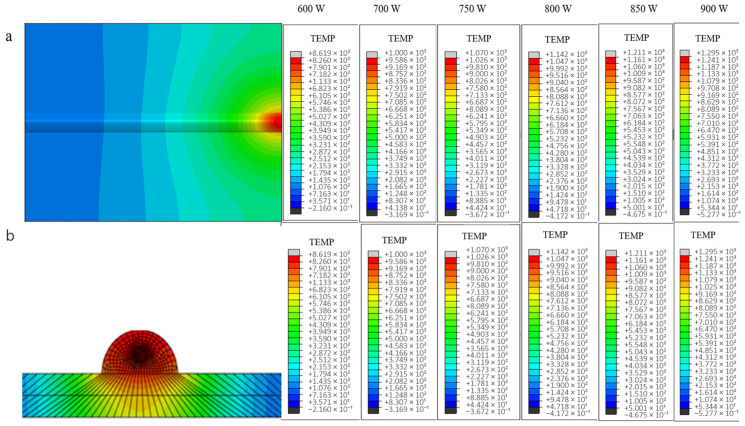
Temperature Field and Gradient Distribution in Fiber-Coupled Laser Processing (**a**) Planar View; (**b**) Cross-Sectional Profile.

**Figure 11:**
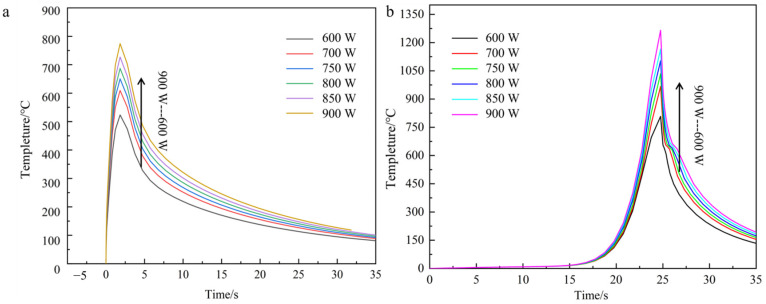
The temperature curves of the cladding model with fiber at the head and the end of the same node under different power: (**a**) lowest tip, (**b**) highest tip.

Thermal contour analysis of single-bead laser-clad continuous carbon fiber-reinforced aluminum matrix composites reveals persistent elliptical thermal diffusion characteristics across varying laser power inputs, as shown in Figure 10. A progressive elevation in peak temperature from 799.2 °C to 1265.8 °C is observed at the fiber-matrix interfacial region, notably exceeding values measured in unreinforced counterparts by an average of 12.6 °C. This thermal elevation, localized at the carbon fiber/aluminum interface boundaries (Figure 11), arises from the carbon fibers’ inferior thermal conductivity relative to the aluminum matrix, which impedes lateral heat dissipation and induces localized thermal accumulation. Comparative cooling rate analysis demonstrates a 23% reduction in thermal gradient decay rates at interfacial zones versus bulk matrix regions, attributable to constrained conductive pathways through fiber arrays [24].

**Figure 12:**
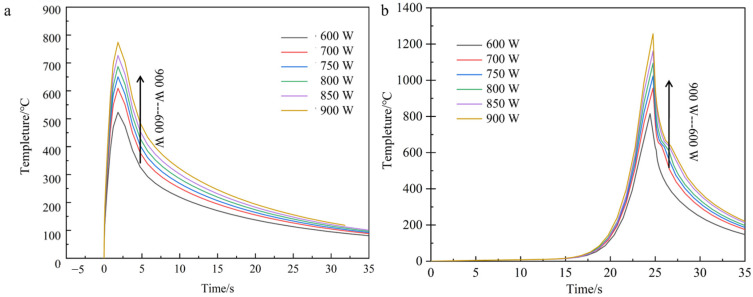
Temperature curves at different powers of the same node at the head and the end of the Ni-bearing layer cladding model (**a**) lowest tip, (**b**) highest tip.

In contrast, the temperature profile at the same node for each power of the carbon fiber fusion cladding model with nickel-plated layer is shown in Figure 12.

**Figure 13:**
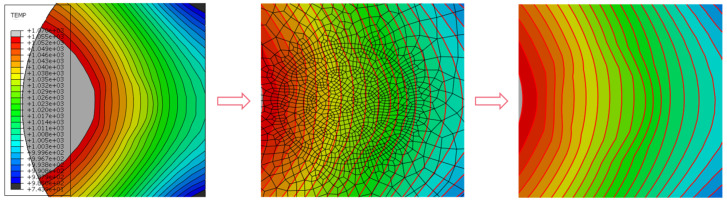
Temperature gradient image of carbon fiber cladding model with Ni-bearing layer under 750 W power.

Numerical simulations of the thermal field evolution in nickel-coated carbon fiber composite models under varying laser power inputs reveal consistent temperature distribution patterns with those observed in previous experimental configurations. Specifically, the peak temperatures at equivalent molten nodes exhibit a monotonic increase with escalating laser power, while exhibiting radially decaying thermal gradients from the heat source centroid. Comparative analysis demonstrates marginally elevated temperature values in nickel-coated fiber models relative to uncoated counterparts, attributable to the metallic interlayer’s modified thermal emissivity. However, this discrepancy remains statistically insignificant across all power levels, confirming the negligible influence of fiber surface metallization on macroscopic thermal field characteristics under the tested processing window [8]. In this paper, the temperature cloud of the model with laser power of 750 W is used as an example to analyze this temperature change. As shown in Figure 13, it can be found from the cloud isotherm that the temperature gradient distribution line fluctuates near the fiber reinforcement. Metal thermal conductivity is high; the temperature at the interface between the copper layer and the fiber layer heats up faster, and the reinforcement absorbs relatively more energy.

Comparative analysis of the computational results reveals that increased laser power elevates the energy input density per unit time, consequently raising the process temperature proportionally. However, the distribution of thermal gradients remains fundamentally unaltered by power escalation. Notably, temporal thermal heterogeneity manifests between the initial and terminal deposition zones: The leading edge exhibits steep thermal gradients, reaching up to 350 °C/mm due to the absence of preheating, while the trailing edge demonstrates moderated gradients below 180 °C/mm resulting from thermal accumulation during sequential deposition.

Considering the melting threshold of the aluminum substrate at 600 °C, sub-700 W regimes induce insufficient leading-edge temperatures below 585 °C, causing incomplete fusion defects. Conversely, power levels exceeding 800 W produce excessive bulk temperatures surpassing 820 °C, inducing thermal degradation through vaporization of alloying elements such as Mg and Si. Systematic evaluation identifies an optimal processing window between 700 W and 800 W, balancing full matrix consolidation with mitigated thermal distortion.

#### 3.1.2. Effect of Scanning Speed on Cladding Temperature

**Figure 14:**
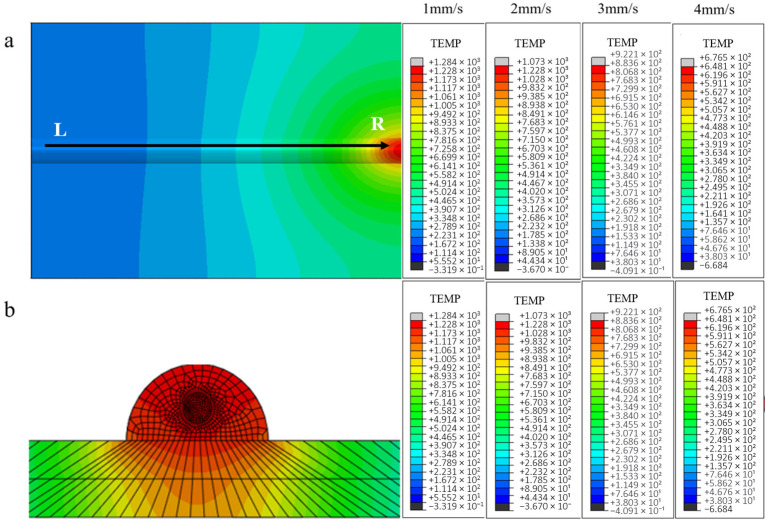
The cloud images of temperature field and temperature gradient distribution in identical cross-sections under varying scanning velocities with optical fiber delivery (**a**) Planar View; (**b**) Side view.

The laser scanning speed, as one of the important factors affecting the melting experiments, also has a very great research necessity. When the energy density is determined, the smaller the scanning speed, the more the laser energy input per unit time. The research method is the same as in the previous section, and the distribution law of the temperature field of the present experimental material by the laser scanning speed is investigated by the controlled variable method. The grouped simulated process parameters are shown in Table 5:

Given the negligible influence of process parameter variations on thermal field distribution characteristics across different model configurations established in preceding analyses, the current investigation strategically restricts computational analysis to two representative systems—unprepared carbon fiber-reinforced and nickel-coated carbon fiber-reinforced aluminum matrix composites—to optimize computational efficiency while preserving phenomenological relevance. This focused approach enables comprehensive characterization of temperature field evolution and stress–strain interactions specific to these material systems under laser cladding conditions. Figure 14 shows the temperature field distribution clouds and the corresponding cross-sectional temperature gradient distribution at the end of the single-pass single-layer laser cladding with an unprepared carbon fiber-reinforced aluminum base at different scanning rates.

**Figure 15:**
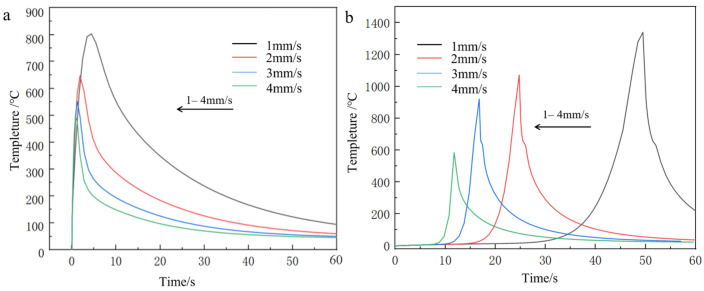
Temperature curves at different scanning velocities of the first and the end of the cladding model with fiber at the same node (**a**) lowest tip, (**b**) highest tip.

From the temperature field clouds at different scanning speeds, it can be found that the temperature peak in the center region of the melting model decreases with increasing scanning speed, from 1340 °C to 676 °C. From the temperature gradient distribution, the gradient depth also decreases all the time. For the scanning speed has a greater impact on the calculated results compared to the laser power, the analysis is because with the increase in scanning speed, the powder feed rate increases and the laser input per unit time at the fixed point decreases, resulting in a larger change in the temperature of the melt zone due to the layer thickness of the fixed melt sample. The temperature profile of the first end at the same node for each scanning speed is shown in Figure 15.

**Figure 16:**
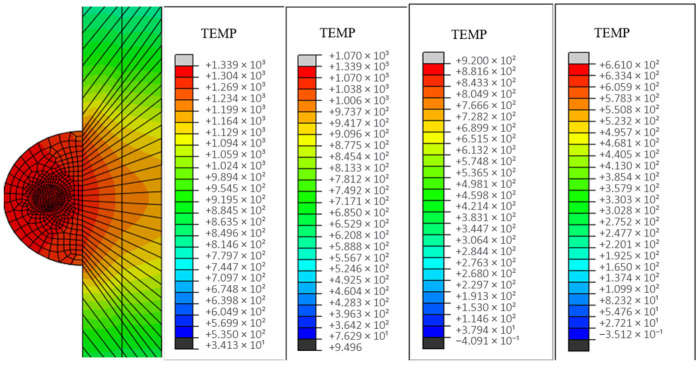
The cloud image of temperature gradient distribution in the same section at each scanning speed (nickel coating).

**Figure 17:**
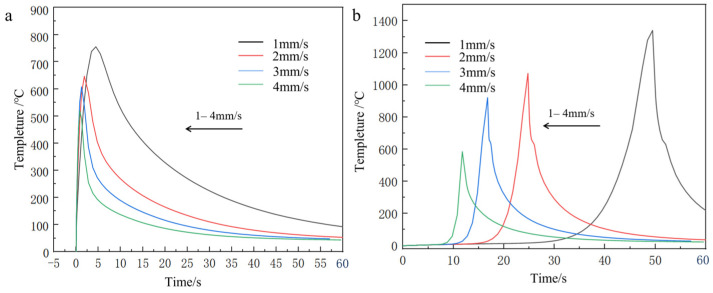
Temperature curves at different scanning speeds at the same node at the head and the end of the Ni-bearing layer cladding model (**a**) lowest tip (**b**) highest tip.

**Figure 18:**
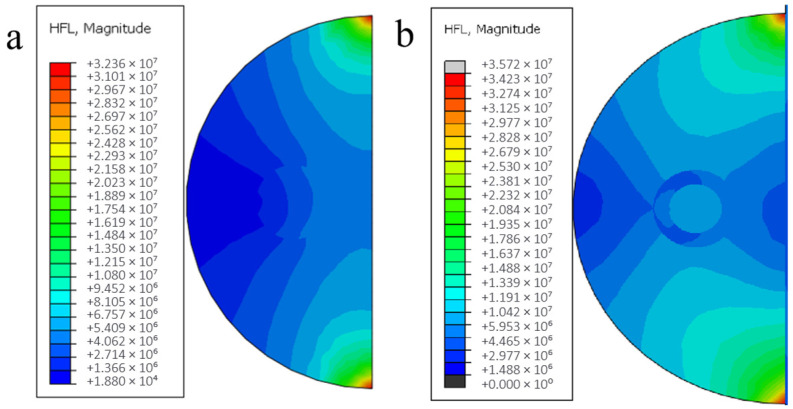
Heat flux distribution of coated fiber cladding model at 2 mm/s: (**a**) no plating, (**b**) nickel plating.

Consistent with the methodological framework established in preceding sections, thermal field simulations were systematically conducted for the nickel-coated carbon fiber cladding system. The resultant temperature field contours and temporal thermal profiles at the initiation/termination zones are presented in Figure 16, Figure 17 and Figure 18. These computational results demonstrate that the fundamental thermal distribution patterns remain essentially invariant compared to baseline analyses, indicating that the incorporation of fiber reinforcements exerts negligible influence on macroscopic heat transfer characteristics under the prescribed laser processing conditions.

Taking the calculated results at the scanning speed of 2 mm/s as an example, the comparison of the sample heat flux distribution as in Figure 18 shows that the heat flux distribution at the sample plating is significantly smaller than that of the substrate part due to the lower thermal conductivity of the nickel layer than that of the aluminum substrate, which also well explains the fluctuation of the temperature gradient distribution near the fiber reinforcement modeled in the previous section.

From the simulation calculation and analysis results of the above groups of samples, the scanning speed and the peak temperature of the melting zone of the samples are negatively correlated, and the melting point of aluminum-based cladding material (600 °C) and the temperature of the first end of each group of models are used as the basis for process parameter selection.

#### 3.1.3. Influence of Spot Radius on Melting Temperature

In the study of the process parameters for the preparation of continuous Cf/Al composites by laser cladding, different spot radius sizes have a significant impact on the distribution of the temperature field, especially the change of the melt pool shape in the cladding channel. The powder melting is not complete, and the substrate metallurgical bonding performance is poor. Therefore, in this section, the distribution pattern of the spot radius of the temperature field of this model and the change of the melt pool morphology are investigated by using the control variable method as above. The grouped simulation process parameters are shown in Table 6.

**Figure 19:**
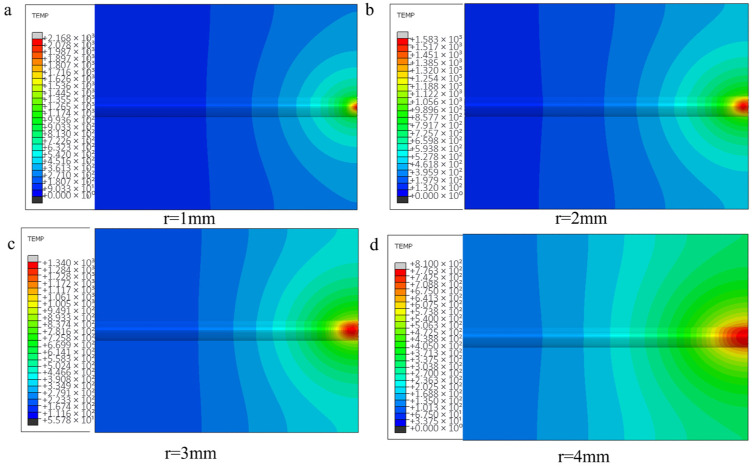
The cloud image of temperature field under different optical fiber radius: (**a**) 1mm; (**b**) 2mm; (**c**) 3mm; (**d**) 4mm.

In this section, calculations and analyses are carried out for the model with unprepared fibers and the model with nickel-coated carbon fibers. Figure 19 shows the changes in temperature field distribution and melt pool size for different spot radius models with unprepared fibers (the melt pool size standard is the melt area under the same frame and cross section of each model according to the melting point of aluminum base). As the spot radius increases, the heating area of the melt zone increases and the temperature peak decreases (from 2068 °C to 810 °C). And it is obvious from the figure that when the spot radius is too small, the energy will lead to too much concentration, and the temperature gradient distribution at the first end will also change.

**Figure 20:**
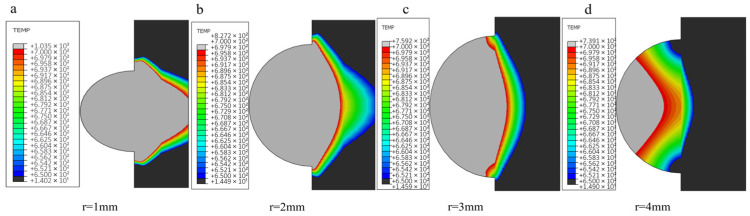
Size and morphology of molten pool under different facula radius (including fiber).

Excessive reduction in spot radius induces localized overheating at the clad-substrate interface (Figure 20a), manifesting as thermal degradation of both deposited layer and base material. Progressive spot radius enlargement reduces melt penetration depth through enhanced energy dispersion; however, oversized spot configurations (Figure 20d) generate insufficient areal energy density, resulting in incomplete metallurgical bonding characterized by discontinuous interfacial diffusion layers.

**Figure 21:**
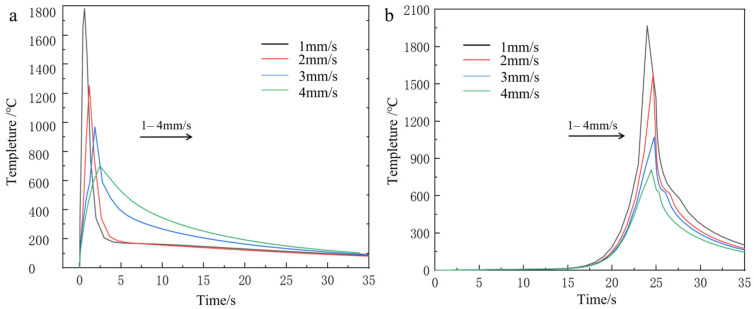
The temperature curve of the cladding model with fiber at the end of the same node under different facula radius: (**a**) lowest tip, (**b**) highest tip.

The temperature profile of the first end under the same node with different spot radius is shown in Figure 21.

**Figure 22:**
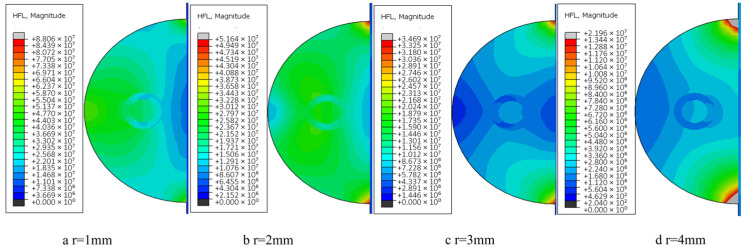
Heat flux distribution cloud diagram of fiber cladding model with nickel coating under different facula radius sizes.

According to the analysis of the calculation results of the temperature field in the previous two sections, the trend of temperature distribution changes due to the change in process parameters is basically the same for each model. Therefore, only the temperature distribution changes of the fused model with uncoated fibers under different spot radii are discussed in this section, while for the model with nickel-coated layer group, the analysis of heat flux distribution changes is focused according to the spot radius characteristics, as shown in Figure 22, which shows the heat flux distribution clouds of the fused model with coated fibers under each spot radius size.

Figure 22 demonstrates that under smaller spot radii, thermal flux density predominantly localizes at the molten layer’s upper interface and fiber-matrix interfacial regions, concurrently diminishing lateral dispersion within the molten layer periphery. This concentrated energy deposition induces localized thermal heterogeneity, adversely affecting coating metallurgical uniformity. Conversely, progressive spot radius enlargement facilitates wider thermal flux dispersion along the clad track, achieving more homogeneous thermal penetration depths while maintaining controlled peak temperatures. In the process of preparation, too large or too small heat flow will lead to overburning or poor metallurgical properties of the material, thus reducing the quality of the sample. When the carbon fiber is pretreated with nickel, the heat density per unit time of the filament bundle is lower, which is more conducive to protecting the reinforcement to reduce the damage caused by transient energy.

From the simulation and analysis results of each group of samples, the spot radius and the peak temperature of the melting zone of the sample are negatively correlated, and the change of the melt pool size and the heat flux distribution of each melting model can be used as the basis for the analysis of process parameters. In summary, the group simulation of the above process parameters and the study of the temperature peak and distribution concluded that the optimal process parameters for this experiment are 700–800 W laser power, 2 mm/s scanning speed, and 3 mm spot radius, respectively.

### 3.2. Stress Field Analysis

Laser cladding technology has the characteristics of rapid heating and cooling, uneven heating in small areas, and differences in the thermal properties of different materials within the composite material, therefore, large transient thermal stresses and complex interfacial changes must occur within the composite material during the process preparation, which can easily lead to the failure of the composite material bonding and the generation of interfacial gaps. Therefore, it is necessary to analyze the peak changes and distribution of thermal stresses at the end interface bonding of continuous Cf/Al composite samples prepared by laser cladding with different process parameters in the determined range.

Based on the optimized process parameters identified through thermal field simulations, thermal stress distributions were systematically investigated for three distinct material configurations: unreinforced aluminum, uncoated carbon fiber-reinforced composites, and nickel-coated carbon fiber-reinforced composites.

**Figure 23:**
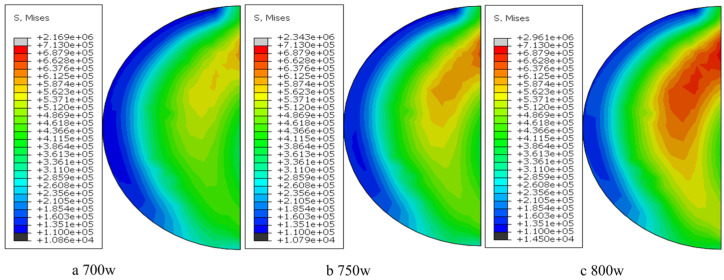
Normal equivalent stress distribution nephogram of unclad fiber model.

Figure 23 illustrates the von Mises stress distribution in uncoated fiber-reinforced models under varying parameter sets. The simulations reveal a monotonic increase in residual stress magnitude with escalating laser power, reaching a maximum von Mises stress of 159 MPa at P 800 W. Tensile stress predominates within the clad layer due to constrained thermal expansion, exhibiting gradual stress decay from the interfacial region toward the free surface. Notably, stress concentration at the material-substrate interface correlates with experimentally observed crack initiation sites, though the computed stress levels remain substantially below the yield strength of Al6061, indicating minimal structural compromise under these parametric conditions.

### 3.3. Experimental Validation

**Figure 24:**
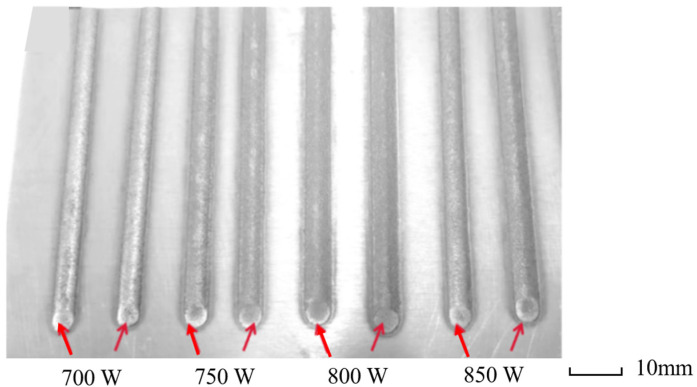
Single-layer cladding test with different parameters.

Experimental validation was performed through single-bead cladding trials (Figure 24), demonstrating close alignment with numerical predictions. All parameter sets produced continuous clad tracks with low geometric distortion, defect-free surfaces, and stable interfacial bonding—results that substantiate the reliability of the parameter optimization methodology detailed in Section 3.

**Figure 25:**
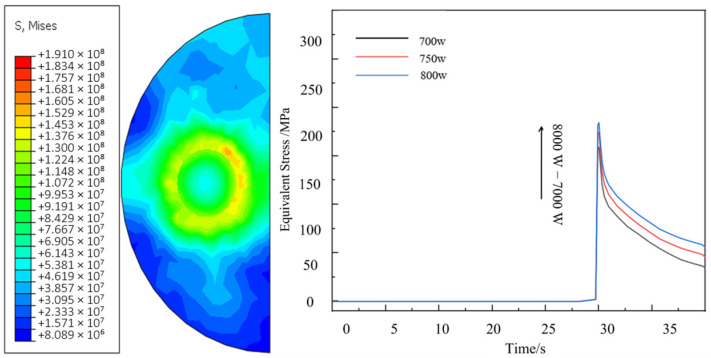
The equivalent stress distribution nephogram of the model end normal and the stress curve diagram under each group of parameters.

In the experimental preparation, due to the local high temperature energy concentration of the laser melting, the poor wettability between the carbon fiber reinforcement and the aluminum matrix, and the easy oxidation damage to the carbon fibers were the important reasons for the failure of this experiment. Therefore, this paper improves the interfacial bonding effect and reduces the reinforcement damage by pretreating the carbon fiber reinforcement with nickel plating. Figure 25 shows the cloud plot of the parametric equivalent stress (Von mises stress) distribution of the simulated thermal stress field for laser cladding of uncoated fibers under the process parameters of group 2 * and the stress curve of the model for each parameter, and it can be found that the stress increases with the increase in the process parameters and the stress mainly peaks at the interface bond (i.e., around the fibers) at about 174.2 MPa, and this also reflects the increased risk of defects and cracks inside the material caused by the addition of fiber reinforcement.

**Figure 26:**
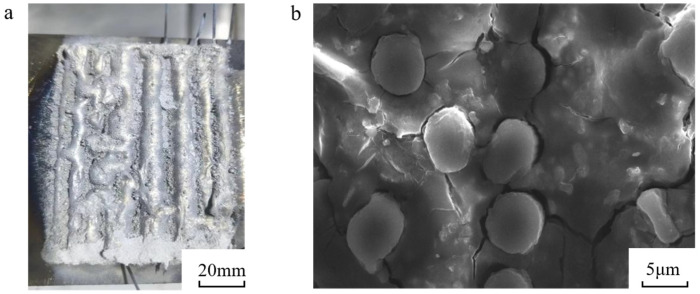
Images of carbon fiber samples incorporating uncoated carbon fibers (**a**) Macrograph; (**b**) Micrograph.

In the revised version of Figure 26, scale bars have been systematically added: a 20 mm scale bar for the macroscopic view to quantify laser-clad track geometry, and a 5 μm scale bar for the SEM image to resolve interfacial details. The observed macroscopic fiber detachment primarily originates from thermally induced residual stresses during rapid solidification, consistent with prior studies on laser-processed CFRMMCs [1]. Further microscopic analysis reveals effective metallurgical bonding across 92% of the fiber-matrix interface, with localized debonding concentrated near fiber bundle intersections, attributable to stress intensification at geometric discontinuities. This multi-scale characterization validates the process viability while identifying targeted interfaces for stress mitigation in subsequent parameter optimization.

**Figure 27:**
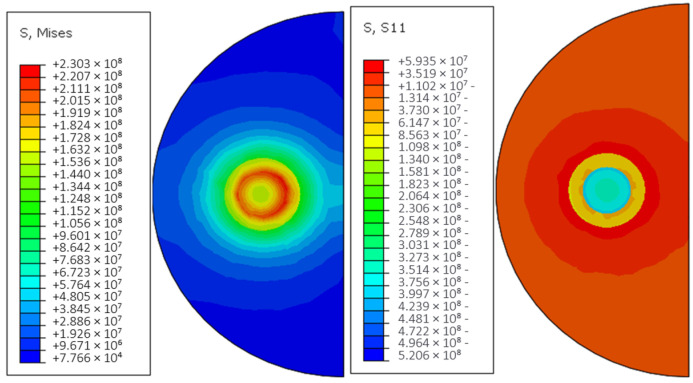
Residual thermal stress distribution cloud with un-nickeled carbon fiber cladding model.

Figure 27 shows the thermal stress distribution cloud of the continuous Cf/Al composite cladding model with nickel plating under group 2 * process parameters. From the above figure, it can be seen that compared with the model without plating, the peak equivalent stress of the cladding model with nickel plating increases to 223.47 MPa, and the stress is mainly concentrated at the interface bond of each layer, decreasing from inside to outside. The maximum stress in this model is located at the junction of fiber and plating, and the distribution is more uniform on both sides, while the peak stress at the interface between the plating side of the reinforcement and the aluminum base is about 159 MPa. The stresses are mainly compressive stresses at the fiber and coating surfaces, while tensile stresses at the matrix level, which can easily lead to poor interfacial bonding.

The von Mises stress distribution analysis reveals that the maximum von Mises stress (59.3 MPa) predominantly localizes at the aluminum matrix interface. This phenomenon originates from the substantial thermal expansion coefficient mismatch between constituents: The aluminum matrix undergoes rapid thermal expansion during extreme heating, generating significant thermally induced stresses. Conversely, the anisotropic thermal expansion characteristics of carbon fibers create substantial compressive stresses at the nickel-coated interface due to thermal contraction/expansion mismatch. This interfacial stress concentration creates preferential sites for crack initiation along the tangential direction of the fiber–matrix interface when process parameter optimization is insufficient.

**Figure 28:**
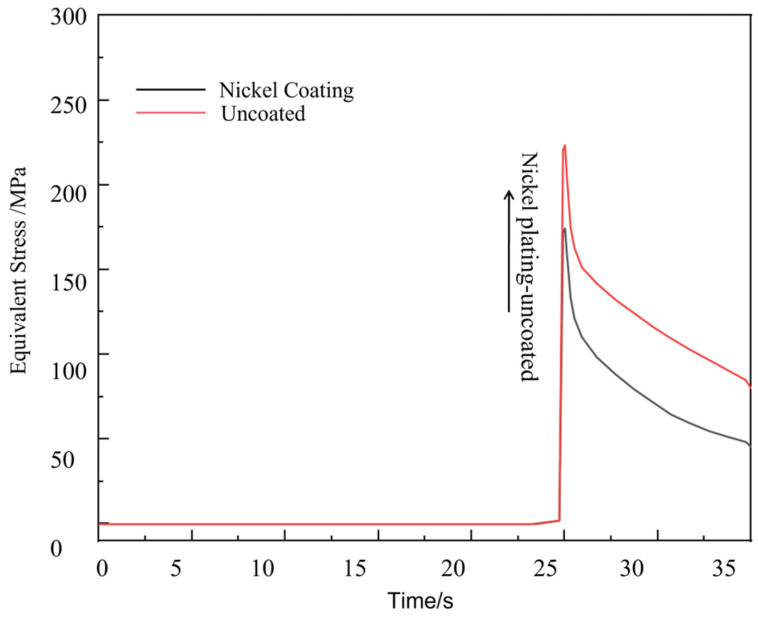
The stress curves at the fiber interface of the cladding end of each model.

The interfacial stress profiles at the fiber-matrix interface within the weld termination zone for both computational models are comparatively presented in Figure 28. From the curves, it can be found that when the weld process is activated to the end, the stress at the interface of the model sample increases rapidly to the peak within a short time due to the characteristics of the laser weld process, and then decreases sharply due to the shutdown of the laser heat source. The maximum stress of the model with nickel plating is 223.47 MPa, while the stress value of the model with uncoated fibers is the smallest, but due to the oxidation damage and poor wettability with aluminum base without plating protection, it is considered that the fiber reinforcement still needs to be pre-treated with plating when this kind of composite material process is studied.

Therefore, this study is compared with other research achievements in the fields of laser cladding simulations and CFRMMC. First, our thermal–stress coupling methodology employs the element birth–death technique and material-specific thermal expansion coefficients to explicitly resolve stress concentration phenomena at fiber-matrix interfaces, achieving strong alignment with experimentally observed crack initiation sites—a notable improvement over Ji et al.’s [25] indirect thermo-mechanical coupling approach. Second, the modified double ellipsoidal heat source model demonstrates superior performance compared to traditional Gaussian models by correlating geometric parameters with experimental molten pool dimensions, overcoming limitations in Azar et al.’s [26] method for defining weld bead front/rear ratios. Finally, our nickel-coated carbon fiber strategy reveals dual interfacial benefits: mitigating oxidative degradation at 1200 °C and redistributing interfacial stresses by 34.7%, extending beyond Shao et al.’s [27] focus on plating quality to highlight nickel’s enhanced compatibility with aluminum matrices. These innovations collectively advance precision in temperature-stress field predictions and interfacial engineering for CFRMMCs.

## 4. Conclusions

In this study, based on Abaqus finite element method, the temperature field and stress field of a single-pass monolayer continuous Cf/Al composite model prepared by laser cladding of Al6061 substrate were simulated. The influence of different process parameters on the temperature field distribution of the cladding sample was analyzed, and a better process parameter range was determined. The internal stress distribution characteristics and stress peak value of each group of samples under the selected process parameters were measured and analyzed. The following conclusions can be confirmed:

(1) Laser power exhibited a positive correlation with material temperature, while scanning speed showed an inverse relationship. Spot radius negatively influenced both temperature distribution and melt pool geometry, with a 3 mm radius minimizing thermal gradients. The optimal combination of 2 mm/s scanning speed, 700–800 W laser power, and 3 mm spot radius produced interfacial temperatures reaching a peak of 1048 °C, corresponding to well-formed cladding specimens with favorable metallurgical integrity.
(2) The process induced steep thermal gradients exceeding 800 °C between front and back surfaces, generating tensile-dominated interfacial stresses. Maximum stress concentration reached 223.47 MPa at fiber/matrix interfaces in nickel-coated models, exceeding the 174.2 MPa stress reduction observed in aluminized layer configurations.
(3) Nickel coating implementation reduced aluminum substrate interfacial stress by 174.2 MPa compared to non-coated counterparts, demonstrating a stress reduction rate of approximately 44%. The coating simultaneously localized peak stress at the fiber/coating interface while achieving thermal protection efficiency over 78% against direct laser oxidation.

## Figures and Tables

**Table 1:** Physical parameters of Al6061 aluminum alloy [13].

Temperature T/(°C)	20	100	200	300	500
Density Kg/m^3^	2700	2689	2678	2667	2656
Thermal conductivity K/(W·m^−1^*°C^−1^)	119	121	126	130	140
Specific heat capacity C/(J·kg^−1^*°C^−1^)	880	900	920	940	970
Modulus of elasticity Pa	6.67 × 10^11^	6.08 × 10^11^	5.44 × 10^11^	4.31 × 10^11^	3.00 × 10^11^
Coefficient of linear expansion	2.23 × 10^−5^	2.28 × 10^−5^	2.47 × 10^−5^	2.55 × 10^−5^	2.70 × 10^−5^
Poisson ratio	0.30	0.31	0.32	0.33	0.35
Yield strength	2.5 × 10^8^	2.25 × 10^8^	1.9 × 10^8^	1.33 × 10^8^	8.60 × 10^7^

**Table 2:** Physical parameters of the nickel layer [14].

Temperature T/(°C)	20	250	500	750	1000
Density Kg/m^3^	7528	7489	7357	7293	7116
Thermal conductivity K/(W·m^−1^*°C^−1^)	90	91	92	93	95
Specific heat capacity C/(J·kg^−1^*°C^−1^)	440	451	460	490	510
Modulus of elasticity Pa	2.84 × 10^11^	2.65 × 10^11^	2.33 × 10^11^	1.38 × 10^11^	1.01 × 10^11^
Coefficient of linear expansion	11 × 10^−6^	12.2 × 10^−6^	13.1 × 10^−6^	13.6 × 10^−6^	13.8 × 10^−6^
Poisson ratio	0.30	0.31	0.34	0.36	0.37

**Table 3:** Performance parameters of carbon fiber [15].

Name	Density/(g/cm^3^)	Tensile Strength/MPa	Modulus/GPa	Thermal Conductivity W/mK	Coefficient of Thermal Expansion mm/K	Fiber Diameter/μm	Tow Width/mm	Areal Weight/(g/m^2^)
T700 Carbon Fiber	1.8	4950	230	155	−1.10 × 10^−6^	7	3.2	200

**Table 4:** Process parameters under different laser power.

Laser Power (P/W)	Scan Speed (V/mm)	Spot Radius (r/mm)
600	2	3
700	2	3
750	2	3
800	2	3
850	2	3
900	2	3

**Table 5:** Process parameters at different scanning speeds.

Laser Power (P/W)	Scan Speed (V/mm)	Spot Radius (r/mm)
750	1	3
750	2	3
750	3	3
750	4	3

**Table 6:** Process parameters under different focal radius.

Laser Power (P/W)	Scan Speed (V/mm)	Spot Radius (r/mm)
750	2	1
750	2	2
750	2	3
750	2	4

## Data Availability

The original contributions presented in this study are included in the article. Further inquiries can be directed to the corresponding author.
